# Practical, Methodological, and Ethical Considerations of User Involvement

**DOI:** 10.1093/geroni/igaa057.2859

**Published:** 2020-12-16

**Authors:** Roger O’Sullivan

**Affiliations:** Institute of Public Health in Ireland, Belfast & Dublin, Northern Ireland, Ireland

## Abstract

Traditionally, older people were seen simply as ‘subjects’ of research but increasingly the case for utilizing the knowledge and insights of older people in planning research, policy, and services is gaining momentum. This presentation explores the nature of user involvement, focusing not just on the practical and methodological considerations, but the ethical aspects, which are often under-discussed. Prof O’Sullivan argues that the increasing focus on user involvement is welcomed but we need to understand and debate how it can be appropriate, meaningful, and beneficial for all involved. Using desk research as well as the results of a web-based survey and semi-structured interviews with researchers, government, and NGO representatives, Prof O’Sullivan will set out the practical as well as the ethical aspects of user involvement. He will recommend how researchers, funders, professional bodies and older people’s organizations and older people themselves can best advance the user involvement agenda. Part of a symposium sponsored by the Patient/Person Engagement in Research Interest Group.

